# Vocabulary, Metalinguistic Awareness and Language Dominance Among Bilingual Preschool Children

**DOI:** 10.3389/fpsyg.2018.01953

**Published:** 2018-10-23

**Authors:** Carmit Altman, Tamara Goldstein, Sharon Armon-Lotem

**Affiliations:** ^1^School of Education at Bar Ilan University, Ramat Gan, Israel; ^2^Department of English Literature and Linguistic, Gonda Multidisciplinary Brain Research Center, Bar Ilan University, Ramat Gan, Israel

**Keywords:** bilingualism, Russian-Hebrew, metalinguistic awareness, dominance, vocabulary size

## Abstract

Awareness of language structure has been studied in bilinguals, but there is limited research on how language dominance is related to metalinguistic awareness, and whether metalinguistic awareness predicts vocabulary size. The present study aims to explore the role of language dominance in the relation between vocabulary size in both languages of bilingual children and metalinguistic awareness in the societal language. It evaluates the impact of two metalinguistic awareness abilities, morphological and lexical awareness, on receptive and expressive vocabulary size. This is of special interest since most studies focus on the impact of exposure on vocabulary size but very few explore the impact of the interaction between metalinguistic awareness and dominance. 5–6-year-old preschool children with typical language development participated in the study: 15 Russian-Hebrew bilingual children dominant in the societal language (SL) Hebrew, 21 Russian-Hebrew bilingual children dominant in the Heritage language (HL) Russian and 32 monolingual children. Dominance was determined by relative proficiency, based on standardized tests in the two languages. Tasks of morphological and lexical awareness were administered in SL-Hebrew, along with measures of receptive and expressive vocabulary size in both languages. Vocabulary size in SL-Hebrew was significantly higher for SL-dominant bilinguals (who performed like monolinguals) than for HL-dominant bilinguals, while HL-Russian vocabulary size was higher for HL-dominant bilinguals than for SL-dominant bilinguals. A hierarchical regression analyzing the relationship between vocabulary size and metalinguistic awareness showed that dominance, lexical metalinguistic awareness and the interaction between the two were predictors of both receptive and expressive vocabulary size. Morphological metalinguistic awareness was not a predictor of vocabulary size. The relationship between lexical awareness and SL-vocabulary size was limited to the HL-dominant group. HL-dominant bilinguals relied on lexical metalinguistic awareness, measured by fast mapping abilities, that is, the abilities to acquire new words, in expanding their vocabulary size, whereas SL-dominant bilinguals and monolinguals did not. This difference reflects the milestones of lexical acquisition the different groups have reached. These findings show that metalinguistic awareness should also be taken into consideration when evaluating the variables that influence vocabulary size among bilinguals though different ways in different dominance groups.

## Introduction

Language dominance among bilingual children can be defined by their relative proficiency in each language, but there is limited research on how language dominance is related to metalinguistic awareness, and whether metalinguistic awareness predicts vocabulary size. The present study aims to explore the role of language dominance in the relationship between vocabulary size in both languages of bilingual children and metalinguistic awareness in the societal language (SL). To achieve this aim, receptive and expressive vocabulary size is tested in both languages of Russian-Hebrew bilingual preschool children who are dominant in one of their languages. This is complemented by measuring metalinguistic awareness in the SL, Hebrew, and by analyzing the relations between vocabulary size and metalinguistic awareness.

### Vocabulary of Monolingual and Bilingual Children

Studies show that bilingual children score below monolingual age appropriate norms when vocabulary size is assessed in only one of their languages (Bialystok et al., 2010; Hoff et al., 2012; Spaulding et al., 2013). For example, Spanish-English bilingual students lag behind monolingual age matched peers in oral language abilities in SL English and in the heritage language (HL) Spanish (Tabors et al., 2003; Páez et al., 2007; Uccelli and Páez, 2007). In particular, English vocabulary skills were limited for children at 4 years of age (Páez et al., 2007), with low levels of vocabulary and gaps between monolingual norms and bilingual children’s scores persisting through first grade (Páez and Rinaldi, 2006). When it comes to vocabulary size in bilinguals’ HL, some studies show poor performance in both receptive and expressive vocabulary (Pearson et al., 1997; Uccelli and Páez, 2007; Bialystok et al., 2010; Verhoeven et al., 2011; O’Toole et al., 2017), while there are other studies that do not show this effect (Umbel and Ki Oller, 1994; Winsler et al., 1999). Moreover, previous findings are not always consistent as to whether a receptive and expressive vocabulary gap (Keller et al., 2015) exists in both languages and if so which factors contribute to its existence. Umbel and Ki Oller (1994), for example, found that Spanish-English bilinguals in first, third, and sixth grade functioned comparably well on the HL Spanish receptive vocabulary test, while SL English receptive vocabulary performance increased with grade level. Furthermore, a receptive-expressive gap was found in a study of 124 Spanish-English bilingual children and 110 monolingual children (mean age = 5;7), for both groups, with a more robust gap amongst the bilinguals, in both languages (Gibson et al., 2012).

These inconsistent results might stem from different factors influencing whether bilingual children perform well or poorly on vocabulary size tests. Therefore, it is important to examine these factors. One often studied factor is exposure. Differences in vocabulary size between bilingual children have often been attributed to variations in the frequency of exposure (Pearson et al., 1997) and, sometimes, to variations in the context of exposure (Bialystok et al., 2010). The vocabulary gap between bilinguals and their monolingual peers is not surprising as children exposed to two languages are likely to hear less of each language during the day than children who are exposed to only one language. Moreover, some words occur in contexts where only one of the languages is used (Fromkin et al., 2007). Consequently, by looking both at English receptive and expressive vocabulary of Spanish-English bilingual children, aged 5–7, Gross et al. (2014) found that bilinguals scored significantly below monolingual children on standardized measures, with bilinguals exposed to SL later lagging behind their peers who were exposed to SL earlier. However, when tested in both languages, the difference in cumulative expressive vocabulary size was no longer significant.

Yet another, less investigated factor is metalinguistic awareness, which might be mediated by language dominance. Metalinguistic awareness builds on earlier linguistic knowledge, which might vary by language dominance, across the two languages of a bilingual child. It is the aim of this paper to assess bilingual dominance and metalinguistic awareness as possible factors that may explain the contradictory results in the literature. The difference between bilinguals and monolinguals in vocabulary size and the gap between expressive and receptive vocabulary further highlight the importance of testing different dominance groups in order to understand the contribution of the relative proficiency in each language in each modality, expressive or receptive (Spaulding et al., 2013).

### Language Dominance Among Bilinguals

The term “language dominance” is used in the literature either for describing the relative proficiency of a bilingual person in the two languages (Gathercole and Thomas, 2009), or for the language the bilingual speaker has been mostly exposed to (Grosjean, 2008). One of the dilemmas which both researchers and language therapists face is how to define dominance (Yip and Matthews, 2007). A most common way is to examine a sample of the child’s productions using one or more performance-based measures and to establish in this way the child’s relative proficiency in his or her two languages. Following Unsworth (2015), this is the way language dominance is defined in the current study. Later age of onset of bilingualism is frequently associated with relative proficiency and more advanced HL outcomes (Hammer et al., 2012; Meir et al., 2016). Yet, age of onset of bilingualism is not necessarily an indicator of dominance, as simultaneous and sequential bilinguals may be found in both the HL-dominant and the SL-dominant groups (Foroodi Nejad and Paradis, 2009). Therefore, the bilingual children in the present study will not be divided into simultaneous and sequential bilinguals, but rather into two dominance groups by their relatively more proficient language.

Language proficiency of bilinguals is often associated with the extent to which vocabulary size in one or both languages meets the norms set for age matched monolinguals (Bialystok et al., 2010). However, bilingual children’s performance may be more varied than monolingual performance as a result of the diversity in their language learning experience (Armon-Lotem et al., 2015). This variation in bilinguals’ performance, often captured in terms of language dominance, might differ as a function of the language skill assessed, resulting in asymmetric linguistic development (Montrul, 2016). While awareness of the formal structure of language has already been studied among bilinguals (Reder et al., 2013), relatively little is known about the association between metalinguistic awareness and vocabulary size in the context of bilingual dominance. It is the aim of this paper to shed light on the relationship between receptive and expressive vocabulary of nouns and verbs in both languages and metalinguistic awareness (morphological and lexical) in the SL among bilinguals, who are dominant in one of their languages.

### Metalinguistic Awareness

Metalinguistic awareness is defined as the ability to distance oneself from the content of speech in order to reflect upon and manipulate the structure of language (Ramirez et al., 2013). Metalinguistic awareness requires the speaker to focus on the structure and form of the language and develops in later stages of language acquisition around the age of 5–6, building on earlier linguistic knowledge (Duncan et al., 2009). Metalinguistic awareness is a set of multiple skills (Bialystok et al., 2014) that are related to the formal aspects of language: phonological, morphological, syntactic and lexical awareness.

Some studies found a statistically significant difference between monolingual and bilingual children on metalinguistic awareness (e.g., Bialystok et al., 2005; Goldstein et al., 2005), pointing out that different skills and tasks might yield different results. For example, Reder et al. (2013) compared 52 French monolingual and 43 French-German bilingual children in first grade, on different metalinguistic skills. While bilingual children outperformed their monolingual peers in morphological compounds and syntactic awareness tasks, no differences were found in morphological affixes and phonological awareness tasks. They argued that due to the phonological similarities between the two languages (French and German), the bilingual children were not required to observe and compare the different linguistic aspects of each language (McBride-Chang et al., 2005). Yet, other studies have shown that bilingual speakers outperform monolingual speakers in metalinguistic awareness tasks (for review see Bialystok, 2001). In particular, in a meta-analysis of 63 studies consisting of 6,022 participants, Adesope et al. (2010) examined the cognitive correlates of bilingualism and found that bilingualism is related to enhanced metalinguistic awareness. The bilingual enhancement observed in the meta-analysis shows the importance of going beyond single studies, which in themselves do not show this effect. However, none of these studies examined the impact of dominance among bilinguals on metalinguistic awareness tasks as the present study intends to do with Russian, the HL, and Hebrew, the SL.

### Metalinguistic Abilities and Vocabulary Size

Vocabulary size is a major factor in language acquisition and as such, it is closely related to metalinguistic skills. On the one hand, vocabulary size is enhanced by metalinguistic abilities and on the other hand, metalinguistic abilities often benefit from a richer vocabulary. Yet, research investigating the metalinguistic abilities in bilinguals focus primarily on phonological awareness and its contribution to reading skills (see example: Carlisle et al., 1999; Ibrahim et al., 2007). Some studies have indeed investigated phonological awareness and vocabulary in bilinguals showing a relationship between phonological awareness and vocabulary (Farnia and Geva, 2011). Children with poorer phonological awareness learned novel and non-novel words less accurately or more slowly (Hu and Schuele, 2005; Hu, 2008). Longitudinally, phonological awareness plays a role when words are relearnt (Hu, 2003) and phonological processing of novel words is based on sublexical representations, which are phonological and unstructured (Marecka et al., 2018).

In comparison, there are hardly any similar studies for morphological and lexical awareness and their association to vocabulary size (Bowey, 1986; Reder et al., 2013). Morphological awareness relates to the ability to manipulate and reflect on morphological units within words (Cheung et al., 2010). It includes the explicit knowledge of the way in which words are built up by combining smaller meaningful units, such as roots, prefixes and suffixes (Guo et al., 2011). Studies have shown that morphological awareness can facilitate word recognition, learning of new words and reading comprehension (Chen et al., 2009; Kraut, 2015).

The importance of morphological awareness for vocabulary learning is well documented in monolingual children (Chen et al., 2012). Nagy et al. (2003) found a strong tie between vocabulary knowledge and morphological awareness, while McBride-Chang et al. (2005) showed that morphological structure awareness and morpheme identification predicted 10% of the variance in vocabulary size. These results underline the importance of examining the impact of different metalinguistic abilities on vocabulary separately in order to understand the variability in vocabulary size (Kuo and Anderson, 2006). Yet, to the best of our knowledge, very little is known about these connections in bilingual contexts in which children acquire vocabulary in two languages and the process might be at a different stage in each language.

Another form of metalinguistic awareness is lexical awareness, which includes conscious consideration of and the ability to manipulate different aspects of lexical competence (Nation, 2008). According to Aşik et al. (2015), lexical competence includes vocabulary size, depth and lexical organization. Nation (2008) argues that lexical awareness can help language learners increase their understanding of the different ways in which vocabulary is used, thus leading, for example, to growth in vocabulary size.

An easy way to measure lexical awareness is fast mapping. Fast mapping refers to the ability of a child to identify the meaning of a novel word after a limited number of exposures (Carey and Bartlett, 1978). It has been observed that growth in vocabulary size is related to fast mapping skills both in the initial stages of word learning (Behrend et al., 2001) and for later acquisition by older children (Braisby et al., 2001). Significant correlations were found between fast mapping performance and vocabulary size scores in early vocabulary acquisition (Kan and Kohnert, 2005; Gray, 2006; Kan et al., 2014), and for older children (ages 4;6–7) with expressive vocabulary scores (Braisby et al., 2001).

Within the developmental lexical principles framework (DLPF) (Golinkoff et al., 1994; Mervis and Bertrand, 1994), fast mapping involves six principles that govern vocabulary acquisition and apply to all languages. The first three include the understanding that words (a) have a reference in the world, (b) can extend to similar referents, and (c) refer to whole objects rather than their parts. These three principles are operative at the onset of lexical acquisition and help in acquiring early vocabulary. The principles which are more related to fast mapping are operative beyond early childhood, in older children and adults (Golinkoff et al., 1992), and are utilized in consciously monitoring the learning of novel words (Ramachandra et al., 2010). These three principles require the: (d) awareness of basic categories for generalization, (e) awareness of constraints on mapping novel names to nameless objects to meet mutual exclusivity, and (f) consideration of the use of conventional names for referents. Fast mapping is an appropriate measure of lexical awareness because the growth in vocabulary size benefits from the latter three principles that operate together. Previous studies have shown the relationship between lexical awareness (measured with this task) and vocabulary size in monolinguals (Behrend et al., 2001; Braisby et al., 2001). Bilinguals also need to apply such constraints when they map novel names to nameless objects. Yet bilinguals also need to learn two labels for the same object, one in each language. In order to abide by the above principles, they should be aware of the differences between the two vocabularies and of translation equivalents. Currently, little is known about the possible interaction between fast mapping and vocabulary size in the case of bilingual children. Of the very few studies of fast mapping and vocabulary size among bilingual children, Kan and Kohnert (2008) do not find such an interaction.

Kan and Kohnert (2008) tested lexical awareness (via fast mapping) and vocabulary size in both the HL (Hmong) and the SL (English) of sequential bilingual children with typical language development (TLD), aged 3–5. In contrast to previous findings with monolingual children, the researchers found that the bilingual children’s fast mapping performance was not related to age or existing vocabulary size in either language. On the other hand, there were significant correlations between vocabulary size and fast mapping across the two languages. For example, fast mapping in English (SL) was negatively correlated with vocabulary size in Hmong (HL), with lower fast mapping abilities in English for children who had larger vocabulary size in Hmong.

According to Kan and Kohnert (2008), this cross-linguistic relation suggests that fast mapping in the SL of bilingual children is not a direct measure of vocabulary size in that language, in contrast to what has been observed in monolingual children. There is, however, a cross-linguistic relationship between fast mapping and vocabulary size in sequential bilinguals – vocabulary size has a negative impact on fast mapping skills in the other language. While the authors made no direct reference to dominance, they suggested that a difference in vocabulary size in either of the languages can perhaps reflect a different stage of language development of sequential bilinguals when compared to monolinguals. Since dominance might be important, but was not considered in this study, we want to replicate the design with participants who are grouped by dominance.

To conclude, although researchers have examined the individual contributions of different metalinguistic abilities to the bilingual lexicon, very few have examined morphological and lexical metalinguistic awareness simultaneously, and even less so with regard to vocabulary size in both languages (McBride-Chang et al., 2005) among bilinguals differing in language dominance.

### Present Study

The present study aims to explore the impact of language dominance on the possible connections between vocabulary size in both languages and metalinguistic awareness in the SL. It is hypothesized that:

(1) Dominance, measured by relative proficiency, will impact vocabulary size in both languages.
(2) Fast mapping used to measure lexical awareness is language neutral and is important for lexical growth (Nation, 2001). It will show a stronger relation to vocabulary size at earlier stages in acquisition and by inference in the less dominant language.
(3) Morphological metalinguistic awareness might be sensitive to language specific knowledge, which requires higher proficiency (Bialystok and Barac, 2012) in the target languages. Therefore, it will show stronger relations with vocabulary size in later stages of acquisition or in the more dominant language.
(4) Fast mapping as a measure of lexical awareness, which is language neutral, is more likely to benefit receptive and expressive vocabulary size than morphological awareness, which is language specific.

In order to test these hypotheses, the study will first examine vocabulary size and metalinguistic awareness separately and then will turn to the relation between the two. Expressive and receptive vocabulary size, as well as morphological awareness and lexical awareness via a fast mapping task, will be tested among HL-dominant and SL-dominant bilingual children with TLD and their monolingual peers. The study is the first to investigate this relation among Russian-Hebrew bilinguals.

The relationships between the two metalinguistic awareness tasks and vocabulary in the context of bilingualism has only rarely been investigated (McBride-Chang et al., 2005). Based on research among monolingual children, correlations are to be expected between the two metalinguistic tasks (morphological and lexical) and vocabulary size in both languages, and in particular between lexical awareness and vocabulary which may be sensitive to dominance.

## Materials and Methods

### Participants

Sixty-eight preschool children with TLD aged 58–78 months (*M* = 68.18, *SD* = 4.66) participated in the present study. Children with different language status formed three language groups: 15 SL-dominant children, 21 HL-dominant bilingual children, and 32 monolingual Hebrew children that served as reference for comparison. Children with hearing impairment, exposure to SL for less than a year or parental concern regarding their child’s language development were excluded from the study. Consent forms were sent to 136 children, out of which eighty were approved. After data was collected, 12 children were excluded from the study after scoring below monolingual and bilingual norms in the language proficiency tests. Inclusion of a bilingual child in the current study was based on a score at or above the provisional bilingual norm (Armon-Lotem and Meir, 2016) in at least one of their languages. Almost all of the participants were born in Israel except for one who was born outside the country and immigrated at the age of 1 year and 10 months. All children attended public preschools in Israel where the language of instruction is Hebrew. Age of onset of bilingualism was determined in months based on parent reports. All children scored above 85 in the “Raven Progressive Matrices” intelligence test (Raven, 1938).

In order to assess children’s language performance in Hebrew, the *Goralnik Screening Test for Hebrew* (Goralnik, 1995) was used. The test includes six subtests: sentence repetition, comprehension, expression, pronunciation, vocabulary, and story-telling sub-tests. The scores are raw scores, with a total of 180 points. The Hebrew cut-off point conforms to former studies of bilingual children in Israel and has provisional bilingual norms (Iluz-Cohen and Armon-Lotem, 2013; Armon-Lotem, 2014; Altman et al., 2016). In order to assess the language performance of the bilingual children in their HL (Russian), the *Russian Language Proficiency Test for Multilingual Children* (Gagarina et al., 2010) was used. The task has a provisional bilingual norm for Russian-Hebrew bilinguals (with a cut-off point of −1.25 SD; Armon-Lotem and Meir, 2016). The raw scores in each screening test were normalized using the provisional norms.

For the present study, dominance was judged based on linguistic performance in two screening tests composed of several sub-tests (e.g., grammar, morphology) testing several domains in each language rather than focusing on a specific domain in order to reflect bilinguals’ performance on a wide range of HL and SL skills. An index of relative proficiency based on the differences between the two language scores, following Cromdal (1999), was calculated and used to determine the bilinguals’ dominance. Relative proficiency was calculated by deducing the normalized HL score from the normalized SL score. This resulted in negative scores for children whose HL scores were higher than their SL scores and positive scores for children whose HL scores were lower than their SL scores. Dominance was measured by a gap of one standard deviation or more between the more proficient and less proficient language as measured by the language screening tests. The index was then used to separate the children into more dominant in the HL or more dominant in the SL. Children’s demographic information appears in Table 1.

**Table 1:** Background and language proficiency information of participants.

Deographic variable	Monolinguals [*N* = 32]	SL-dominant bilinguals [*N* = 15]	HL-dominant bilinguals [*N* = 21]	*df*	*F*
Age in months	67 (4.3)	69.33 (4.67)	69.66 (4.75)	2,65	2.61
Hebrew proficiency (z-score)	0.19 (0.81)	0.46 (0.65)	−1.71 (1.47)	2,65	27.4^∗∗∗^
Russian proficiency (z-score)	NA	−2.67 (1.79)	0.57 (0.88)	1,34	51.52^∗∗∗^
Age of onset	NA	14.33 (19.28)	41.38 (18.59)	1,34	17.95^∗∗∗^
Length of exposure	NA	54 (21.63)	28.66 (16.80)	1,34	15.64^∗∗∗^

*^∗∗∗^p < 0.001.*

ANOVAs conducted to examine language proficiency differences between the bilingual dominance group showed differences in Hebrew *F* (1,34)=28.61, *p* < 0.001 and Russian proficiency *F* (1,34)=51.52, *p* < 0.001. Additional ANOVAs show significant differences in terms of age of onset (AoO) as well as in length of exposure (LoE), *F* (1,34) = 17.95, *p* < 0.001 and *F* (1,35) = 20.45, *p* < 0.001, respectively. These differences were expected since AoA and LoE are known to influence dominance. A one-way ANOVA investigating whether there are difference between the three language status groups showed a significant different *F* (2,65) = 27.4, *p* < 0.001. A Bonferroni *post hoc* test yielded significant differences in Hebrew proficiency between monolinguals and HL-dominant bilinguals (*p* < 0.001) and between SL-dominant bilinguals and HL-dominant peers (*p* < 0.001) as expected due to the relative dominance in the languages, with no difference between the SL-dominant group and monolinguals. It should also be noted that no age differences were detected among the three groups *F* (2,65) = 0.66, *p* > 0.05.

### Measures

#### Cross Linguistic Lexical Task (CLT)

Children’s vocabulary size in both languages was assessed with the Hebrew version of the LITMUS CLT-task (Haman et al., 2015; Altman et al., 2017; O’Toole et al., 2017), and the Russian version of the LITMUS CLT task^1^ (Gagarina and Nenonen, 2017, Unpublished). Both versions of LITMUS CLT contain four separate subtests, measuring receptive, and expressive nouns and verbs separately. Receptive vocabulary is tested through a picture selection task with four pictures and expression through a naming task. Each subtest is composed of 32 items scored as correct or incorrect using the classification of responses described for LITMUS CLT (Haman et al., 2015). The final score is assigned to each subset as a percentage of correct responses out of 32.

#### Morphological Awareness Task

A morphological awareness task was developed for Hebrew following McBride-Chang et al. (2005). The task included 14 test items that test consonantal root awareness (Hebrew being a Semitic language) and lexical compound awareness. For each item, the child is presented with two pictures of homophones that sound the same but have different meanings, and sometimes, different roots. The test items contained either two homophone verbs, two homophone nouns, or a homophone noun and verb. The examiner names each one orally. The child is then presented with the target item; a word or a lexical compound derived from one of the meanings of the homophone. The child is asked to choose the picture that corresponds best to the meaning of the target item. This requires knowledge that the words share the same root. The prompt in this task was: “Which of the pictures is more related to the word”…?. For example, the child is shown two pictures: “*or”* (light) and “*or”* (skin), and is asked to match correctly the word “*teura*” (lighting) to the target picture depicting “*or”* which shares the same root. A second example is “*yalda”* (a girl) and “*yalda*” (gave birth) – and the lexical compound “*erec moledet*” (place of birth). In this case, both pictures share the same root with the target item, but only one shares the meaning.

Each item includes an open question asking the child to explain his answer (“why did you choose this answer?”) in order to examine in a more qualitative manner the children’s responses and what they could reveal about their metalinguistic ability. A certain concern was raised that this task may tap into semantic association knowledge due to the use of pictures. Nevertheless, the pictures were considered necessary in order to administer and adapt this task to preschool children. The final score was assigned as a percentage of correct responses out of 14. The overall reliability of this task is α = 0.54.

#### Lexical Awareness Task

A fast mapping task was used to test lexical awareness (Kan and Kohnert, 2008). Novel bisyllabic non-words (CVCVC, e.g., *renil, tumof, pamig, xemog*) were presented to the children. The novel words were not easily associated with any existing referent in either language in order to minimize the possibility for phonological or semantic associations. A PowerPoint presentation was used to present children with an undersea creature who was teaching them the names of undersea objects. In the first stage, the child was simultaneously presented with four pictures on the screen and was asked to recognize a novel object among three known distractors (*“Where is the pamig?”*). The novel referent was presented among known objects to measure mutual exclusivity. After the child identified the object, she got a confirmation (*Right, this is the pamig*), or correction (*Are you sure?* *I think this is the pamig*), and was asked to repeat the word (*Can you say pamig*?). In total, the child was exposed to the word three times and was asked to repeat it once. In the next stage, the child was asked to identify the novel word with a referent that had the same shape but a different color among a second set of objects, two known and two novel. The child was asked again “*Where is the pamig?”* This measured receptive generalization skills, which are important since the child has to distinguish between the new word and other new concepts not known to him. This procedure was repeated four times with different items. To make it fun for the children a memory game followed in which the children were asked to name all new objects. One point was assigned to each correct response. Due to a high correlation between the mutual exclusivity and the generalization measures (*r* = 0.753, *p* < 0.001), only the generalization measure, which is the closest indication of the child’s acquisition of the new word, was chosen to measure the child’s lexical awareness skill, yielding a maximum score of four.

### Procedure

The children were assessed individually in their preschool or in their homes in a private room for two sessions unless a specific child required more time. The children participated voluntarily and each child received a small reward (a sticker or a toy) at the end of each session as a token of appreciation to encourage their continuous collaboration. All responses were both audio-recorded and manually recorded on a response sheet. Parental consent was obtained, during which parents answered a short background questionnaire concerning demographic and language acquisition information, and the children’s oral assent was secured. The study was approved by the university IRB and by the Israeli Ministry of Education.

### Data Analysis

The information obtained from the four parts of the LITMUS CLT task in each language was calculated as a percentage of correct responses. The size of expressive and receptive vocabulary was calculated by combining the nouns and verbs and calculating the percentage of correct responses. The choice to present the results for both receptive and expressive vocabulary reflects the reported gap between the two, especially among bilingual children (Gibson et al., 2012), and the possibility that this gap is a reflection of the need to suppress the competition between the two languages in a naming task which could be sensitive to dominance. Consequently, a series of multivariate analyses of variance as well as ANOVAs were conducted to compare between bilingual dominance groups on HL and SL vocabulary size measures and between bilingual dominance groups and monolinguals on SL vocabulary size.

The metalinguistic awareness tasks were calculated separately as a percentage of correct responses (morphological and lexical). Relative proficiency was used as a measure of dominance for the hierarchical regression analyses. Following a comparison of the metalinguistic awareness measures across the bilingual dominance groups and the monolingual children, hierarchical regression was conducted introducing relative proficiency first, then the metalinguistic awareness tasks and finally the interactions between relative proficiency and metalinguistic awareness. The choice of hierarchical regression was motivated by the desire to explore the relative contribution of each predictor. The hierarchical regressions were conducted separately for receptive and expressive vocabulary in both the HL and the SL of all bilingual children as one group. As we used hierarchical regression with 5 predictors the model could be prone to overfitting. Thus, in order to confirm the results we further used linear regressions to test only the two metalinguistic predictors for each of the dominance groups separately as well as for the monolinguals, allowing us to tease apart their relative contribution to vocabulary size.

## Results

### Vocabulary Measures

**FIGURE 1:**
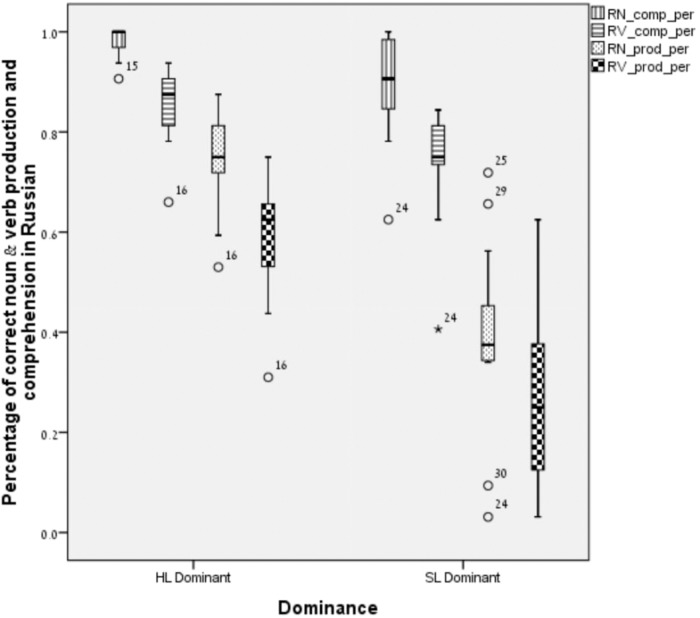
Noun and verb production and comprehension in HL-Russian.

**FIGURE 2:**
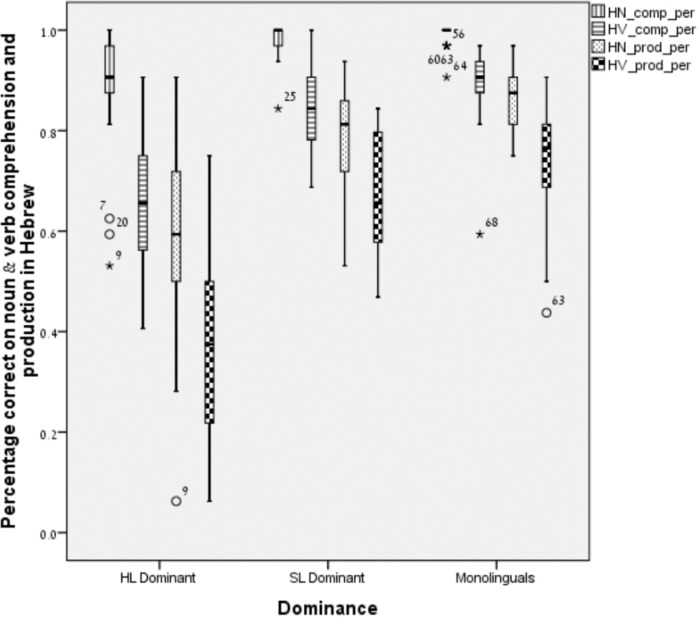
Noun and verb production and comprehension in SL-Hebrew.

In order to explore whether vocabulary size is different in the two dominance groups, descriptive results on both receptive and expressive abilities of children on verbs and nouns in their HL (Russian) and SL (Hebrew) are presented. Table 2 presents a comparison of the HL-dominant bilingual children to the SL-dominant bilingual children. Monolingual data is presented for SL only. Figures 1, 2 present the group differences in HL and SL, respectively.

**Table 2:** Vocabulary size via receptive and expressive vocabulary of nouns and verbs in the HL (Russian) and SL (Hebrew).

	HL-Russian vocabulary		SL-Hebrew vocabulary		
	SL-dominant	HL-dominant	SL-dominant	HL-dominant	Monolingual
Noun receptive	0.90 (0.12)	0.98 (0.03)	0.97 (0.04)	0.88 (0.14)	0.99 (0.02)
Verb receptive	0.76 (0.11)	0.85 (0.07)	0.85 (0.10)	0.66 (0.13)	0.89 (2.16)
Noun expressive	0.50 (0.24)	0.70 (0.16)	0.78 (0.12)	0.58 (0.21)	0.86 (0.06)
Verb expressive	0.36 (0.21)	0.55 (0.16)	0.68 (0.13)	0.35 (0.20)	0.73 (0.1)

Table 2 shows that vocabulary size mirrors the dominance level of the two groups. The children’s performance was better in the language in which they were dominant in terms of both receptive and expressive vocabulary.

For HL-Russian, a one-way MANOVA, with nouns and verbs receptive and expressive vocabulary scores in Russian as dependent variables, and language groups (SL-dominant vs. HL-dominant bilinguals) as an independent variable, was conducted. A significant multivariate effect was found for Language groups, *F* (4,31) = 17.47, *p* < 0.05; Wilks’ λ = 0.3, *η^2^* = 0.69, such that HL-dominant bilinguals outperformed SL-dominant bilinguals in receptive and expressive vocabulary in Russian (HL). Moreover, univariate testing indicated significant differences between the two language groups in each of the LITMUS CLT tasks: In the noun receptive task, *F* (1,34) = 12.76, *p* < 0.01, *η^2^* = 0.27; in the verb receptive task, *F* (1,34) = 15.21, *p* < 0.001, *η^2^* = 0.31; in noun expression, *F* (1,34) = 64.48, *p* < 0.001, *η^2^* = 0.65; and in verb expression, *F* (1,34) = 42.48, *p* < 0.001, *η^2^* = 0.55. That is, there were significant differences between the two groups on all four vocabulary measures, with HL-dominant bilinguals outperforming SL-dominant bilinguals in receptive and expressive nouns and verbs in HL/Russian, as can be seen in Figure 1.

Likewise, for SL-Hebrew, an initial two-way MANOVA was conducted, with nouns and verbs receptive and expressive scores as dependent variables and language group (monolingual, SL-dominant, HL-dominant) as independent variables. Significant multivariate effect for language group, *F* (8,124) = 10.37, *p* < 0.001: Wilks’ λ = 0.36, *η^2^* = 0.4. A follow-up Bonferroni analysis showed that the average test score of monolinguals and dominant SL bilinguals was statistically higher than that of HL-dominant bilinguals in all four categories (*p* < 0.001). There were no significant differences between the monolinguals and the SL-dominant bilinguals. Moreover, univariate testing indicated significant differences between the two language groups in each of the LITMUS CLT tasks in Hebrew (SL): In the noun comprehension, *F* (1,34) = 7.11, *p* < 0.05, *η^2^* = 0.17; in verbs comprehension, *F* (1,34) = 20. 94, *p* < 0.001, *η^2^* = 0.38; in nouns expression, *F* (1,34) = 11.53, *p* < 0.01, *η^2^* = 0.25; and in verbs expression, *F* (1,34) = 32.52, *p* < 0.001, *η^2^* = 0.49. That is, there were significant differences between the two groups on all four vocabulary measures, with SL-dominant bilinguals outperforming HL-dominant bilinguals in receptive and expressive nouns and verbs in SL/Hebrew, as can be seen in Figure 2. Finally, there was a gradual pattern in all groups where the highest scores were found in noun receptive vocabulary followed by verb receptive vocabulary and only then did the expressive vocabulary follow with children performing higher on noun expressive vocabulary than on verb expressive vocabulary.

### Metalinguistic Awareness Measures

Metalinguistic awareness was measured in Hebrew. Descriptive results comparing the three groups’ performances in the two metalinguistic awareness tasks (morphological and lexical) are presented in Table 3.

**Table 3:** Morphological and lexical metalinguistic awareness tasks.

Metalinguistic awareness	HL-dominant	SL-dominant	Monolinguals
Morphological	0.62 (0.11)	0.71 (0.13)	0.72^∗^ (0.15)
Lexical	0.68 (29)	0.77 (0.29)	0.75 (0.21)

*^∗^p < 0.05 for the difference between HL-dominant bilinguals and monolinguals.*

In order to examine whether there were differences between the three groups in the two metalinguistic awareness tasks, a one-way ANOVA was conducted. Significant differences were revealed in the morphological awareness task *F* (2,65) = 3.74, *p* < 0.05. A *post hoc* Bonferroni analysis revealed that monolinguals outperformed HL-dominant bilinguals (*p* < 0.05), with no significant differences between monolinguals and SL-dominant bilinguals or between HL-dominant and SL-dominant bilinguals. No Univariate effect was found for language groups in the lexical awareness task, *F* (2,64) = 0.70, *p* > 0.05.

### Metalinguistic Awareness and Vocabulary Size

The major aim of the paper was to explore the relative contribution of dominance measured by relative proficiency, lexical and morphological metalinguistic awareness and the interaction between dominance and metalinguistic awareness in the SL-Hebrew to receptive and expressive vocabulary size in Hebrew in comparison to Russian. A hierarchical regression analysis was conducted with all five predictors, introducing relative proficiency first, followed by the metalinguistic awareness measures, and finally the interaction between relative proficiency and metalinguistic awareness.

### SL-Hebrew Receptive Vocabulary

Table 4 presents a summary of the hierarchical regression analysis for variables predicting receptive lexicon in SL-Hebrew.

**Table 4:** Summary of hierarchical regression analysis for variables predicting receptive vocabulary size (*N* = 35).

	Model 1			Model 2			Model 3		
Variable	*B*	*SE B*	*ß*	*B*	*SE B*	*ß*	*B*	*SE B*	*ß*
RelProf	0.024	0.005	0.666^∗∗∗^	0.024	0.005	0.604^∗∗∗^	0.081	0.026	2.209^∗∗^
LexM				0.128	0.052	0.306^∗^	0.158	0.047	0.376^∗∗^
MorphM				0.044	0.136	0.045	0.099	0.125	0.101
RelProf × LexM							−0.059	0.018	−1.290^∗∗^
RelProf × MorphM							−0.022	0.032	−0.409
*R^2^*	0.444			0.535				0.662
*F*	26.363^∗∗∗^			11.879^∗∗∗^			11.348^∗∗∗^		

*RelProf, Relative proficiency; LexM, Lexical metalinguistic awareness; MorphM, Morphological metalinguistic awareness. ^∗^p < 0.05, ^∗∗^p < 0.01, ^∗∗∗^p < 0.001. One child was excluded from the analysis since he was missing lexical awareness scores.*

The hierarchical regression analysis shows that relative proficiency alone (Model 1) significantly predicted the size of receptive vocabulary [ß = 0.666, *t*(34) = 5.134, *p* < 0.001]. Model 1 explained 42% of the variance in the size of receptive vocabulary [*F* (1,33) = 26.363, *p* < 0.001]. When metalinguistic awareness measures are added in Model 2, the model significantly predicted the size of receptive vocabulary [ß = 0.604, *t*(33) = 4.293, *p* < 0.001 for relative proficiency, ß = 0.306, *t*(33) = 2.458, *p* = 0.02 for lexical metalinguistic awareness], explaining together 49.7% of the variance [*F* (3,31) = 11.879, *p* < 0.001]. Morphological metalinguistic awareness made no significant contribution. When the interactions are added in Model 3, the new model significantly predicted the size of receptive vocabulary [ß = 2.209, *t*(32) = 3.084, *p* = 0.004 for relative proficiency, ß = 0.376, *t*(32) = 3.349, *p* = 0.002 for lexical metalinguistic awareness, and ß = −1.290, *t*(32) = −3.273, *p* = 0.003 for the interaction between relative proficiency and lexical metalinguistic awareness], explaining together 60.3% of the variance [*F* (5,29) = 11.348, *p* < 0.001]. Model 3 suggests that while relative proficiency and lexical metalinguistic awareness are positively related to the size of receptive vocabulary, the interaction between them is negatively related to the size of receptive vocabulary. Morphological metalinguistic awareness and the interaction between relative proficiency and morphological awareness have no significant contribution.

Due to the small number of bilingual participants, the hierarchical regression used above with five predictors is prone to overfitting. Therefore, we further conducted a linear regression for each dominance group in which only the two metalinguistic awareness measures were introduced as predictors. A similar linear regression was conducted for the monolingual group to provide a baseline for comparison. Table 5 presents a summary of a linear regression analysis for the two variables predicting receptive vocabulary size for HL-dominant and SL-dominant bilinguals as well as monolinguals.

**Table 5:** Summary of linear regression analyses for variables predicting receptive vocabulary size for HL-dominant, SL-dominant bilinguals, and monolinguals.

	HL-Dominant [*N* = 21]			SL-Dominant [*N* = 14]			Monolinguals [*N* = 32]		
Variable	*B*	*SE B*	*ß*	*B*	*SE B*	*ß*	*B*	*SE B*	*ß*
LexM	0.270	0.074	0.658^∗∗^	−0.061	0.052	−0.322	0.017	0.032	0.100
MorphM	0.314	0.192	0.296	0.112	0.117	0.267	0.063	0.045	0.252
*R^2^*		0.435			0.161			0.083	
*F*		6.928^∗∗^			1.057			1.306	

*LexM, Lexical metalinguistic awareness; MorphM, Morphological metalinguistic awareness. ^∗^p < 0.05, ^∗∗^p < 0.01, ^∗∗∗^p < 0.001. One child was excluded from the analysis since he was missing lexical awareness scores.*

The linear regression showed that for HL-dominant bilinguals lexical metalinguistic awareness significantly predicted the size of receptive vocabulary [ß = 0.658, *t*(20) = 3.623, *p* = 0.002], while morphological metalinguistic awareness does not contribute. Lexical metalinguistic awareness explained 37.2% of the variance in the size of receptive vocabulary [*F* (2,18) = 6.928, *p* = 0.006]. For the SL-dominant group and the monolingual group, no predictors were found to contribute.

### SL-Hebrew Expressive Vocabulary

Similar results were observed for the expressive vocabulary. Table 6 presents a summary of the hierarchical regression analysis for variables predicting the expressive vocabulary in SL-Hebrew.

**Table 6:** Summary of hierarchical regression analysis for variables predicting expressive vocabulary size (*N* = 35).

	Model 1			Model 2			Model 3		
Variable	*B*	*SE B*	*ß*	*B*	*SE B*	*ß*	*B*	*SE B*	*ß*
RelProf	0.047	0.008	0.723^∗∗∗^	0.040	0.008	0.617^∗∗∗^	0.123	0.043	1.905^∗∗^
LexM				0.257	0.081	0.347^∗∗^	0.296	0.078	0.399^∗∗^
MorphM				0.216	0.219	0.126	0.304	0.207	0.177
RelProf × LexM							−0.069	0.030	−855^∗^
RelProf × MorphM							−0.047	0.052	−0.507
*R^2^*		0.522			0.644			0.704	
*F*		36.081^∗∗∗^				18.717^∗∗∗^		13.823^∗∗∗^	

*RelProf, Relative proficiency; LexM, Lexical metalinguistic awareness; MorphM, Morphological metalinguistic awareness.^∗^p < 0.05, ^∗∗^p < 0.01, ^∗∗∗^p < 0.001.*

The hierarchical regression analysis shows that relative proficiency alone (Model 1) significantly predicted the size of the expressive vocabulary [ß = 0.723, *t*(34) = 6.007, *p* < 0.001]. Model 1 explained 50.8% of the variance in the size of the expressive vocabulary [*F* (1,33) = 36.081, *p* < 0.001]. When metalinguistic awareness measures are added in Model 2, the model significantly predicted the size of the expressive vocabulary [ß = 0.617, *t*(33) = 5.015, *p* < 0.001 for relative proficiency, ß = 0.347, *t*(33) = 3.189, *p* = 0.003 for lexical metalinguistic awareness], explaining together 61% of the variance [*F* (3,31) = 18.717, *p* < 0.001]. Morphological metalinguistic awareness made no significant contribution. When the interactions are added in Model 3, the new model significantly predicted the size of the expressive vocabulary [ß = 1.905, *t*(32) = 2.846, *p* = 0.008 for relative proficiency, ß = 0.399, *t*(32) = 3.802, *p* = 0.001 for lexical metalinguistic awareness, and ß = −0.855, *t*(32) = −2.321, *p* = 0.028 for the interaction between relative proficiency and lexical metalinguistic awareness], explaining together 65.3% of the variance [*F* (5,29) = 13.823, *p* < 0.001]. Model 3 suggests that while relative proficiency and lexical metalinguistic awareness are positively related to the size of expressive vocabulary, the interaction between them is negatively related to the size of expressive vocabulary. Morphological metalinguistic awareness and the interaction between relative proficiency and morphological awareness have no significant contribution.

Due to the small number of bilingual participants, the regressions used above with five predictors is prone to overfitting. Therefore, we further conducted a simple linear regression for each dominance group in which only the two metalinguistic awareness measures were introduced as predictors. A similar linear repression was conducted for the monolingual group to provide a baseline for comparison. Table 7 presents a summary of the simple regression analyses for the two variables predicting expressive vocabulary size for HL-dominant and SL-dominant bilinguals as well as monolinguals.

**Table 7:** Summary of linear regression analyses for variables predicting expressive vocabulary size for HL-dominant, SL-dominant bilinguals, and monolinguals.

	HL-Dominant [*N* = 21]			SL-Dominant [*N* = 14]			Monolinguals [*N* = 32]		
Variable	*B*	*SE B*	*ß*	*B*	*SE B*	*ß*	*B*	*SE B*	*ß*
LexM	0.402	0.125	0.596^∗∗^	0.087	0.099	0.245	−0.007	0.063	−0.021
MorphM	0.699	0.323	0.401^∗^	0.233	0.220	0.293	0.055	0.091	0.114
*R^2^*		0.411			0.157			0.013	
*F*		6.285^∗∗^			1.028			0.185	

*LexM, Lexical metalinguistic awareness; MorphM, morphological metalinguistic awareness. ^∗^p < 0.05, ^∗∗^p < 0.01, ^∗∗∗^p < 0.001.*

The linear regression showed that for HL-dominant bilinguals both lexical metalinguistic awareness and morphological metalinguistic awareness significantly predicted the size of expressive vocabulary [ß = 0.596, *t*(20) = 3.216, *p* = 0.005 and ß = 0.401, *t*(20) = 2.162, *p* = 0.044, respectively]. The model explained 34.6% of the variance in the size of expressive vocabulary [*F* (2,18) = 6.285, *p* = 0.009]. For the SL-dominant group and the monolingual group, no predictors were found to contribute.

### Comparing SL-Hebrew Receptive and Expressive Vocabulary

**FIGURE 3:**
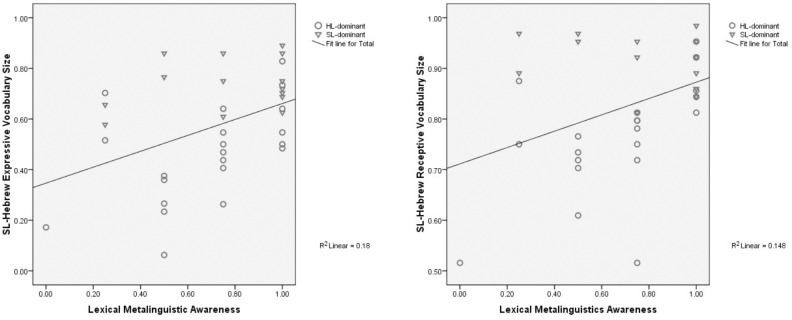
The impact of lexical metalinguistic awareness on receptive and expressive vocabulary size.

The similarity in the impact of lexical metalinguistic awareness on receptive and expressive vocabulary size is further demonstrated in the scatter plots in Figure 3.

### HL-Russian Receptive and Expressive Vocabulary

The contribution of lexical metalinguistic awareness to vocabulary size in SL-Hebrew is in sharp contrast to the findings for HL-Russian vocabulary size. Similar regression analyses conducted with HL-Russian receptive and expressive vocabulary size as the dependent variables, showed that only relative proficiency (which is positive for SL-dominant and negative for HL-dominant, by definition) negatively predicted vocabulary size in HL-Russian. Dominance measured by relative proficiency was the only predictor, explaining over 50% of the variance in receptive vocabulary, and over 70% of the variance in the expressive vocabulary. The metalinguistic awareness measures and the interactions were introduced in models 2 and 3, respectively, and had insignificant contribution.

## Discussion

The present study explored the possible connections between vocabulary size and different metalinguistic awareness abilities among bilingual children of different dominance groups and monolingual children with TLD. The first hypothesis that dominance, measured by relative proficiency, will impact vocabulary size in both languages was confirmed. Dominance groups differed in terms of vocabulary size. As expected, HL-dominant bilinguals outperformed SL-dominant bilinguals on SL-Russian receptive vocabulary. By contrast, SL-dominant bilinguals and monolinguals outperformed HL-dominant bilinguals on receptive and expressive vocabulary size in SL-Hebrew. For metalinguistic awareness, no difference was found among the groups with one exception: monolinguals outperformed HL-dominant bilinguals on the morphological awareness tasks. When focusing on the different dominance groups, the linear regression showed that metalinguistic awareness abilities predicted vocabulary size only for the HL-dominant group, confirming the second hypothesis. Morphological metalinguistic awareness predicted vocabulary size only for expressive vocabulary among the HL-dominant group refuting the third hypothesis. The hierarchical regression analyses showed, that dominance, as well as lexical metalinguistic awareness and the interaction between the two, predicted receptive and expressive vocabulary size. Morphological metalinguistic awareness did not predict vocabulary size. This confirms the fourth hypothesis. Finally, no effect of metalinguistic awareness on HL, Russian vocabulary size, was observed for either group.

### Receptive and Expressive Vocabulary

The results of the LITMUS CLT vocabulary task are in line with previous findings (e.g., Bialystok et al., 2010), with dominant HL bilinguals lagging behind their age-matched dominant SL and monolingual peers on all four vocabulary measures in SL (Hebrew), but outperforming their SL-dominant peers on all four vocabulary measures in HL (Russian). The lack of differences between monolinguals and SL-dominant bilinguals in vocabulary size is not surprising, considering the relative exposure to SL of the SL-dominant group (*M* = 54 months), most of whom are simultaneous bilinguals. The regression analysis further showed that dominance measured by relative proficiency was the best predictor of receptive vocabulary among bilingual children confirming the first hypothesis.

Moreover, all children in this study had more difficulty with the expressive tasks than with the receptive tasks, as is consistent with previous literature (e.g., Gibson et al., 2012), with a bigger gap between receptive and expressive vocabulary size in SL-Hebrew for the bilingual groups (especially amongst HL-dominant) as opposed to the monolingual group. The expected receptive-expressive gap reflects the difference between the two processes. Receptive vocabulary that taps into lexical knowledge is less sensitive to language dominance than expressive vocabulary, that taps on lexical knowledge and its retrieval. This reflects the impact of dominance on more demanding processes (expression). The gap is consistently smaller in the dominant language (Russian in the HL-dominant bilinguals and SL-Hebrew in the SL-dominant bilinguals) than in the weaker language. Moreover, SL-dominant bilinguals perform like monolinguals. The sensitivity of lexical access to dominance suggests that the competition between the two linguistic representations of each concept is influenced by the relative proficiency in each language. While the receptive vocabulary is similar, the smaller gap in the dominant language suggests that linguistic representation in this language is more readily available in lexical access.

### Metalinguistic-Awareness Abilities

Children demonstrate metalinguistic awareness in later stages of language development, around the age of 5–6, after gradually mastering the structure of the language, accumulating vocabulary, and developing efficient access to words and concepts (Duncan et al., 2009). The present study shows no differences between the three groups of 6-year-olds in terms of metalinguistic awareness, except for one instance where monolinguals did significantly better than HL- dominant bilinguals on a morphological awareness task. Russian and Hebrew have very distinct morphological features, especially in word formation. Russian word formation highly relies on concatenative morphology (Shevelov, 1957), while Hebrew word formation mostly uses non-concatenative morphology (Berman and Bolozky, 1978; Aronoff, 1994). Previous studies suggested that morphological awareness requires high proficiency in a given language (Bialystok and Barac, 2012); thus, morphological awareness in SL-Hebrew requires high proficiency in SL-Hebrew. The finding that monolinguals outperformed the HL-dominant bilinguals on the morphological awareness task is in line with this assumption.

As the morphological task in this study depended on knowledge of SL-Hebrew derivational morphology, and knowledge of Hebrew derivational morphology requires, in turn, extensive knowledge of vocabulary, the limited Hebrew vocabulary size of HL-dominant children can be responsible for the gap in morphological metalinguistic awareness. A possible support for this explanation comes from the performance of the SL-dominant bilinguals. The SL-dominant bilinguals often patterned with the monolinguals showing a significant difference from HL-dominant bilinguals. By contrast, for morphological awareness, they showed no significant differences from monolinguals as well as HL-dominant bilinguals, performing in between the two groups.

An explanation of the relatively limited morphological awareness abilities of HL-bilinguals could be their relatively low length of exposure to the SL, a variable that has great impact on language proficiency for bilingual children (e.g., Chondrogianni and Marinis, 2011). It is possible that HL-dominant bilinguals, who are often sequential bilinguals, did not have sufficient exposure (12–34 months) to their SL (Hebrew) in order to develop high morphological awareness in this language. Yet, the absence of a significant difference from the SL-dominant group that has longer exposure undermines this explanation. A definite conclusion on this is hampered by the small sample of children in the SL-dominant bilingual group (*N* = 15) and the considerable variance in the length of exposure of the group (*M* = 54, *SD* = 28.66), which might have resulted in the lack of statistical differences between SL-dominant bilinguals and the other two groups.

Finally, the lack of difference between the groups in lexical awareness might have to do with the task selected for the present study. Lexical awareness was assessed through a fast mapping task. Fast mapping requires children of the age tested to consult their vocabulary when encountering a new word in order to meet the requirement of assigning a novel label to a novel object on the one hand and abide by conventionality on the other. Fast mapping resembles the situation often encountered in language learning by monolinguals (mapping a novel word form to a novel object). In bilingual language learning, the novel word in the SL is mapped onto a known object with a known label in the HL and does not follow mutual exclusivity. The lack of difference between the groups suggests that bilingual experience does not impact fast mapping as a measure of lexical metalinguistic awareness.

### The Relation Between Vocabulary Size and Metalinguistic Awareness

Better metalinguistic skills are expected to positively impact the acquisition of the SL. Hierarchical regression analyses tested the impact of dominance, measured by relative proficiency, lexical metalinguistic awareness, morphological metalinguistic awareness and the interaction of the two with dominance on the size of receptive and expressive vocabulary in both the HL and the SL. These analyses showed that beyond the significant impact of dominance, lexical metalinguistic awareness, but not morphological awareness, influenced vocabulary size. Despite the gap between receptive and expressive vocabulary, the impact of metalinguistic awareness was similar in the two modalities. The contribution of lexical metalinguistic awareness to vocabulary size among bilingual children suggests that bilinguals, like monolinguals, rely on fast mapping in expanding their vocabulary size. More specifically, the principles that are operative beyond early childhood for consciously monitoring the learning of novel words (Ramachandra et al., 2010) were found to be related to expanding the lexicon in the SL. The awareness of constraints on mapping novel names to nameless objects to meet mutual exclusivity, seems to help in mapping novel words in the SL to objects, even if they already have a name in the HL. Likewise, the consideration of the use of conventional names for referents, seems to not block the process of mapping a novel name in one language to familiar objects that already have a conventional name in the other. This suggests that the utilization of the principles of fast mapping is sensitive to the language that is acquired. Having a label for an object in one language does not interfere with acquiring a new label in the other.

Our findings even suggest that experience with fast mapping, which is language neutral, helps in increasing the size of the lexicon. Morphological awareness, by contrast, was found to make little contribution, especially when the interaction between dominance and metalinguistic awareness was considered in the equation. These findings suggest that the language specific nature of morphological awareness tasks makes it impossible to rely on experience in one language in learning new words in the other.

Moreover, the significant contribution of lexical metalinguistic awareness to vocabulary size was limited to the SL-Hebrew, and was not observed in the HL-Russian. Experience with fast mapping, which is language neutral, seems to be transferred from the HL to the SL and helps in increasing the vocabulary size in the SL only. This asymmetry reflects the different phase each group is in for vocabulary acquisition in the two languages. A large number of the bilinguals in this study had a smaller vocabulary size in SL-Hebrew compared to HL-Russian. This suggests that they need to learn new vocabulary items at a more rapid speed in SL-Hebrew than in the HL-Russian. In such a case, better fast mapping skills can become useful.

This latter proposal is supported by the findings of the linear regression that lexical awareness was found to influence SL vocabulary size only in the HL-dominant group. While higher relative proficiency and greater lexical metalinguistic awareness was related to greater receptive and expressive vocabulary in SL-Hebrew, there was also an interaction between proficiency and lexical awareness. This interaction showed that the relationship between lexical awareness and vocabulary size was stronger for participants with lower proficiency. This supports the assumption that better lexical awareness, and in particular better fast mapping skills, predicts growth in vocabulary size, in different ways for different relative proficiency levels. In particular, this confirmed our second hypothesis that fast mapping which is important to lexical growth will show a stronger relation to vocabulary size at earlier stages in acquisition, that is, in the less dominant language.

These results were further confirmed by the linear regression conducted when focusing on each dominance group separately. For the different dominance groups, the regression analyses revealed that children rely on this metalinguistic ability if the SL is their less dominant language. The task used for lexical awareness predicts success in acquiring a larger vocabulary among the least proficient group, strengthening the above explanation, and showing the importance of introducing relative proficiency into the equation. The relationship between Hebrew vocabulary size and lexical awareness ability was found only among HL-dominant bilinguals, but not for the other groups. The absence of such a relationship among the SL-dominant bilinguals is reminiscent of Kan and Kohnert (2008) findings. There, they tested the relationship between lexical awareness (via fast mapping) and vocabulary size in both the HL and the SL (English) of sequential bilingual children with TLD, aged 3–5 and found that there were no significant correlations between vocabulary size and fast mapping across the two languages. Our SL-dominant bilingual children seem to be at the same stage of vocabulary acquisition as the children in Kan and Kohnert (2008) study were. As the HL-dominant bilinguals are at the earlier stage of vocabulary acquisition, they still rely on these abilities, while the SL-dominant bilinguals and monolingual children are beyond this phase and therefore present a different profile. In sum, our findings suggest that metalinguistic awareness might have a different effect on vocabulary size at different levels of acquisition, which is consistent with the previous literature that shows different cognitive mechanisms operating at different stages of language acquisition (Gathercole et al., 1992; Hu, 2008).

Our findings for morphological metalinguistic awareness can also shed light on the question of whether metalinguistic awareness depends on the stage of language acquisition of SL that each group is at. Metalinguistic awareness might be limited by restricted formal linguistic knowledge in a particular language (Bialystok et al., 2014) and the stage in which each group is at in their language acquisition of SL. There are reasons to assume that the outcomes of this study, and in particular the negative relation observed among HL-dominant children between morphological awareness and their HL-vocabulary size, are related to their limited exposure to Hebrew morphology used in the relevant metalinguistic tasks. A task that will add measures of metalinguistic abilities in the HL will enable more definite conclusions.

To conclude, this study highlights the importance of considering dominance when studying language abilities and metalinguistic awareness among bilinguals. This is important in order to provide a more accurate account of the impact of bilingualism and better our understanding of the contribution of the relative proficiency in each language in each modality (expressive and receptive) and of metalinguistic awareness to vocabulary growth among bilinguals. A strong similarity was found between SL-dominant and monolingual children in SL vocabulary size while HL-dominant bilinguals lagged behind. By contrast, HL-dominant bilinguals outperformed SL-dominant bilingual on HL vocabulary size. The novelty of this study lies in the finding that the relation between metalinguistic awareness and vocabulary size were different in the two dominance groups. The HL-dominant group presented an earlier phase in the acquisition of the SL, in which vocabulary size in the SL is sensitive to lexical awareness, while vocabulary size in the HL hinders the development of morphological awareness in the SL. HL-dominant bilinguals relied on lexical metalinguistic awareness, measured by fast mapping abilities in expanding their vocabulary size, whereas SL-dominant, like monolinguals, did not. This shows that lexical awareness is important for word learning at more initial stages of vocabulary acquisition. While many studies show the relevance of length and amount of exposure to vocabulary size, the present study shows that metalinguistic awareness should also be taken into consideration, and might make different contributions in different dominance groups.

## Ethics Statement

This study was carried out in accordance with the recommendations of ethics guidelines of Bar Ilan University’s IRB. The protocol was approved by the Bar Ilan University IRB as well as by the ethics committee at the Ministry of Education in Israel. All parent gave written informed consent and children gave their assent orally in accordance with the Declaration of Helsinki.

## Author Contributions

CA, TG, and SA-L were responsible for the conception, analysis, and interpretation of the work. The paper was drafted and revised to include intellectual content by CA, TG, and SA-L. CA is accountable for the integrity and accuracy of the work. The paper was approved for publication of content by SA-L.

## Conflict of Interest Statement

The authors declare that the research was conducted in the absence of any commercial or financial relationships that could be construed as a potential conflict of interest.

## Footnotes

1 The overall reliability of this task is α = 0.961 for the Hebrew version and α = 0.956 for the Russian version.
